# Digital Microscopy Assessment of Angiogenesis in Different Breast Cancer Compartments

**DOI:** 10.1155/2013/286902

**Published:** 2013-09-01

**Authors:** Anca Haisan, Radu Rogojanu, Camelia Croitoru, Daniela Jitaru, Cristina Tarniceriu, Mihai Danciu, Eugen Carasevici

**Affiliations:** ^1^Emergency Medicine Department, Gr. T. Popa University of Medicine and Pharmacy, 700115 Iasi, Romania; ^2^Emergency Medicine Department, Saint Spiridon Hospital, 700115 Iasi, Romania; ^3^Pathophysiology and Allergy Research Department, Medical University of Vienna, 1090 Vienna, Austria; ^4^TissueGnostics GmbH, 1020 Vienna, Austria; ^5^Pathology Department, County Emergency Hospital, 610136 Piatra Neamt, Romania; ^6^Laboratory of Molecular Biology, Regional Institute of Oncology, 700483 Iasi, Romania; ^7^Department of Anatomy, Gr. T. Popa University of Medicine and Pharmacy, 700115 Iasi, Romania; ^8^Pathology Department, Gr. T. Popa University of Medicine and Pharmacy, 700115 Iasi, Romania

## Abstract

*Background/Aim*. Tumour angiogenesis defined by microvessel density (MVD) is generally accepted as a prognostic factor in breast cancer. However, due to variability of measurement systems and cutoffs, it is questionable to date whether it contributes to predictive outline. Our study aims to grade vascular heterogeneity by comparing clear-cut compartments: tumour associated stroma (TAS), tumour parenchyma, and tumour invasive front. *Material and Methods*. Computerized vessel area measurement was performed using a tissue cytometry system (TissueFAXS) on slides originated from 50 patients with breast cancer. Vessels were marked using immunohistochemistry with CD34. Regions of interest were manually defined for each tumour compartment. *Results*. Tumour invasive front vascular endothelia area was 2.15 times higher than that in tumour parenchyma and 4.61 times higher than that in TAS (*P* < 0.002). Worth to mention that the lymph node negative subgroup of patients show a slight but constant increase of vessel index in all examined compartments of breast tumour. *Conclusion*. Whole slide digital examination and region of interest (ROI) analysis are a valuable tool in scoring angiogenesis markers and disclosing their prognostic capacity. Our study reveals compartments' variability of vessel density inside the tumour and highlights the propensity of invasive front to associate an active process of angiogenesis with potential implications in adjuvant therapy.

## 1. Introduction

Quantification of tumoral tissue vascularization has become important to scientific research since Folkman's revolutionary idea that no tumoral tissue can grow more than 2 mm without vascularisation [1–5]. Despite the scientific effort, there is still no standardized protocol to count and analyze neoangiogenesis [6]. The most frequent method to assess angiogenesis is microvessel density (MVD) by visual count of stained blood vessels performed under high magnification light microscopy [7, 8]. Because of interobserver variability, many studies use at least 2 experts to count the vasculature [9–11]. One limiting factor of this method comes from the fact that counting is done only on few areas assessed as being among the most vascularized spots. These few spots are selected by the human experts, thus adding inherent interuser variability and poor intrauser reproducibility. The few identified “hot spots” have a low statistical significance when compared to the analysis of the entire section, and the obtained results may not reflect the whole reality [12]. Even the widely used Chalkley eye piece graticule is depending on human experience [13–23].

Nowadays computer analysis of endothelial area became more frequently used in tumoral angiogenesis studies. However, digitization instrumentation, analysis input, their processing workflow, and performed measurements vary from one study to another, and none was yet adopted as a standard procedure for vasculature assessment. Moreover, to our best knowledge, none of them investigated intercompartment comparisons of vasculature parameters [24–29]. 

Computer analysis of virtual slides was used in several studies [30–33] in which angiogenesis was assessed in the whole tissue section. Since the research purpose remains to discover the most aggressive form of tumour and to predict its development, we believe that identification of some domains of interest of tumour such as tumor associated stroma (TAS), tumoral area/parenchyma (T), and invasive front (IF) and quantification of angiogenesis in every compartment may be a more reliable predictor of tumoral outcome and a useful indicator for adjuvant therapy.

Our aim is to assess vasculature in distinct tumoral compartments in an objective manner using a reproducible automated method. The various tumoral compartments shall be manually marked up on virtual slides by a human expert as follows: invasive front, tumour area, and tumour associated stroma.

## 2. Materials and Methods

### 2.1. Patients and Tissues

We analyzed 50 samples of carcinoma of patients between 37 and 70 years old (mean age 57), diagnosed with breast invasive carcinoma, NST (invasive ductal carcinoma, NOS). The women were without any hormonal or chemotherapy before the surgical resection. For each patient, we gathered additional information like medical pathological records; morphological description, TNM classification, histological grade, ER, PR, HER2/neu expression, molecular subtypes, and various correlations were investigated statistically. 

### 2.2. Immunohistochemistry

Paraffin embedded tumor blocks were cut (5 *μ*m sections) and mounted on adhesive slides. Additional sections were performed for subsequent negative and positive controls. Sections were deparaffinised in xylene baths then rehydrated in graded alcohols to water antigen retrieval was made by HIER method using a steamer (90°C for 30 minutes), in high pH Dako retrieval solution; endogenous peroxidase activity was quenched by 0.3 H_2_O_2_ at room temperature, 10 minutes. After Tris-buffered saline (TBS) washing, blocking solution was used (Protein Block Serum-Free, Ready to use, Dako). The slides were washed with TBS again. Test lot was incubated over night at 4 Celsius degrees after applying primary antibody rabbit monoclonal ab81289 [EP373y] anti= CD34 Abcam, at a 1 : 200 dilution. The negative control lot was incubated in the same manner but using monoclonal mouse IgG1 antibody instead of the primary antibody. Second day we applied on both lots biotinylated link and detection complex, LSAB-kit Dako. Developing reaction was made with DAB chromogen and, after that, counterstained with Mayer's haematoxylin, fixed in tap water, dehydrated in alcohol and xylene, clarified, mounted, and cover slipped.

### 2.3. Scanning Procedure

Samples were digitized and analyzed with TissueFAXS 3.5 (TissueGnostics Gmbh, Vienna, Austria) which included both the scanner as well as the cytometry analysis packages (TissueQuest and HistoQuest). The system consisted of a Zeiss Imager Z2 microscope equipped with a 3 Megapixel area scan colour camera Pixelink PL-623 CF. The motorized stage from Maerzhauser had an 8-slide insert easing the batch scan. The white light was delivered by a VISLED lamp based on LED technology which ensured a stable reproducible intensity over the entire study. The computer used for analysis was an HP Z400 running on an Intel Xeon W3565 processor at 3.2 GHz under Windows 7 32 bit.

The image acquisition phase was done with a 10x magnification objective. Proper microscope settings were checked every day following Koehler illumination procedures described in Zeiss Imager manuals. The camera sensor was aligned to the stage so that the angle between their axis was less than 0.01 degrees, thus avoiding systematic image alignment errors. TissueFAXS 3.5 scanning software was set to store the image tiles in JPEG format with a 95% quality index. The virtual slides were realized by enabling an image overlap of 15% while the integrated algorithm realigned the fields of view by using the overlapping content. This fine-tuning step corrected minor stage errors (up to 2 *μ*m as reported by Maerzhauser, Germany). Every scanning project acquired a nonsaturated white image for shading compensation. Lamp voltage was set to 6 V so that the microscopic visual assessment using the oculars can be done without changing settings other than the light path. Camera exposure time value was manually set on a white image so that the background had a value of 235 on all red, green, and blue channels. Automatic white balance was performed at the same spot. Gamma enhancements were set to OFF, imposing a linear quantitative behaviour of the image intensities. All parameters related to lamp and camera were stored in a TissueFAXS intensity profile for easy use during scanning of all slides of the study, thus ensuring a consistent nonvariable quality of images. The scanned slides used 60 GB on a NTFS formatted hard disk; backups were done using external USB hard drives. Total scanned area over the entire set of 50 patients was 47957 mm^2^.

### 2.4. Definition of Regions of Interest

An initial contextual user analysis phase included visual assessment of the virtual slides from a pathological point of view. Thus, the sections were investigated for locations of tumour area (parenchyma), tumour associated stroma (TAS), and invasive front. The tumour area was identified having an irregular stellated outline pattern, including epithelial tumour cells describing ducts, nests, and cords. The invasive front was perceived as the interface between the periphery of tumour and the adjacent breast tissue. We observed that the breast tumour growth pattern is characterized most frequently by infiltrating and widespread dissection of normal tissue with loss of clear boundary between tumour and host tissues. In addition, we sometimes noted at high magnification the particular aspect of invasive front, with discontinuous small aggregates or single, isolated tumour cells, pattern also known as “tumour budding” [34–36]. The microenvironment surrounding or including the malignant cells is defined as tumour associated stroma. Studies of the desmoplastic reaction in the stroma established that this is a very useful histological prognostic parameter for patients with breast cancer [37]. We used the same criteria for classification of fibrotic cancer stroma as Ueno did in [37], such as: mature (composed of mature, fine, elongated collagen fibers, with multi-layer fibrocytes), intermediate (broad bands of collagen keloid-like were intermingled with mature collagen fibers) and immature fibrotic stroma (randomly orientated collagen bundles surrounded by abundant myxoid stroma). 

For each one of these domains, 1–3 sites were selected and marked for analysis using standard regions of interest (ROI) tool by adding predefined circular shaped ROIs of 1 mm diameter. Each of these ROIs had contours highlighted in green, blue, or red, as they belonged to stroma, tumour, or invasive front, respectively. Setting of such coloured highlighting decreased user errors and improved the time spent on visual assessment and secondary opinion analysis during reaching interexpert agreement as well as during postanalysis checks. ROIs selections were done so that difficult image areas were avoided. Folded tissue or with mechanical disrupted morphology generated by cutting, air bubbles within mounting medium or major staining artefacts were disregarded from the analysis.

**Figure 1:**
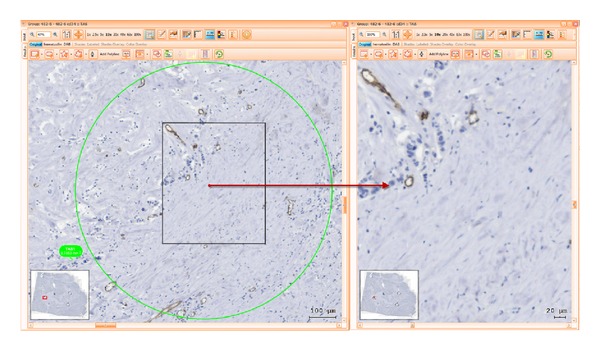
Selecting an ROI of tumour associated stroma (TAS) immunomarked with CD34-green circle, having at the same time an overview of the whole sample, and zoom in of ROI.

**Figure 2:**
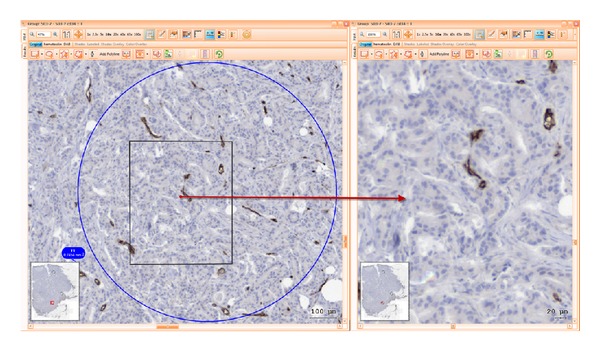
ROI of tumour (T) area stained with CD34, overview of whole sample, and zoom in target area.

**Figure 3:**
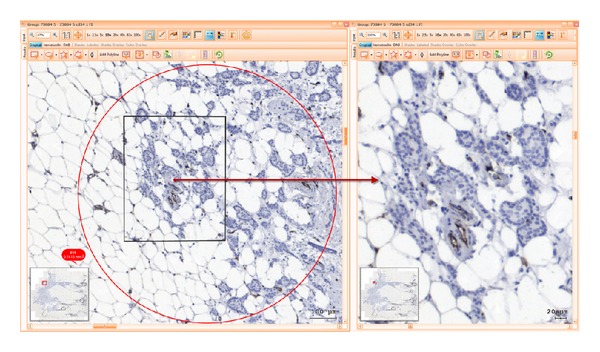
Invasive front (IF) selection area stained with CD34, overview of whole sample, and zoom in marked area.

Following these rules, two-phase ROI definition was performed. During the first phase, 2 pathologists (CC, MD) independently selected the ROIs. A second review phase included multiple consensual meetings in order to confirm, comment, or change ROI sites upon common agreement in all samples (Figures 1, 2 and 3).

*Print Screens of ROIs During Computer Analysis.* See Figures 1, 2, and 3.

### 2.5. Measurement Procedure

The computer analysis was done using HistoQuest 3.5 cytometry software. TissueFAXS virtual slides were imported into HistoQuest analysis projects, with reusing shading corrections and image tile overlapping information. The software splits the color of RGB image into marker-specific channels using an integrated colour separation method named single reference shade. This approach can separate Hematoxylin and CD34-DAB stains into their optical density (OD) counterparts after a training procedure which involves pointing with the mouse pixels for each of the two stains. The system does not require preparation of separate samples stained only with one of the two markers as it can compensate also for mixtures of stains used as training data. The colour separation method computes an abundance map for each marker, extracting for each pixel the amount of Hematoxylin and CD34-DAB, respectively. This approach allows the assessment of CD34-DAB pixel intensities independently of other existing counterstain. Having the CD34-DAB abundance maps, simple thresholding can be applied to extract positive areas. Although the software allows automatic controlled thresholding, the preferred method included setting a manual threshold of the CD34-DAB OD for all samples and quantifying endothelial area (EA) using total area measurement option. A manual iterative search of the proper threshold was performed by looking at the image results showing overlays of contours on top of original images of several samples. The interactive threshold selection features of HistoQuest, as well as the possibility to apply the settings on small test regions, allowed for a visual confirmation of selected parameters during the iterative search. A HistoQuest marker profile was created to save all colorimetric and thresholding parameters and was used in all analysis projects of the study. 

The analysis of all projects containing both definitions of the ROIs within the domains and the analysis parameters was performed using the batch analysis module of HistoQuest. This allowed automatic unsupervised quantification of the entire data set, which took about 4 hours running on all the available 4 cores of the processor.

### 2.6. Validation of Endothelial Area Detection

Having all projects analyzed, a visual validation of proper EA identification was performed. Each project was reopened, and overlays of vessel contours on the colour images were assessed. Blood vessels which were not identified by the system due to too weak stain intensities were manually indicated using Manual Correction Tool -> Add Event. On the other side, as CD34 is known to be expressed also in fibroblast [38], identified objects which did not show typical morphology of blood vessels (fibroblasts, staining artefacts, and high background) were manually removed from analysis using Manual Correction Tool -> Delete Events. From all 50 patients, only 4 virtual slides required manual intervention on the automatic identification of EA. 

### 2.7. Extraction of Quantitation Data

Measurement results were exported from the validated analyzed projects using batch export module which generated a single excel sheet containing all relevant patient data: total analyzed area (AA) and total endothelial area (EA) of each domain of each patient. Derived measurements were computed directly in Excel, one of them being the relative endothelial area (REA) for each domain (REA percent = 100 ∗ EA/AA). Statistical analysis was performed in SPSS 19 using the data from exported excels.

## 3. Results 

### 3.1. Endothelial Area Quantitation

**Figure 4:**
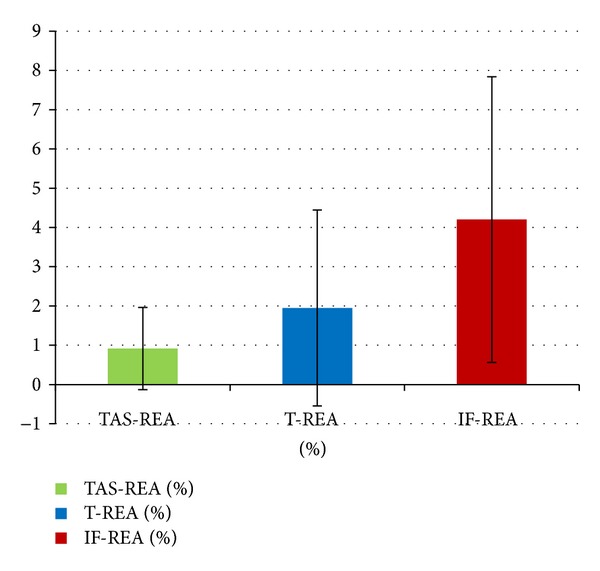
Average REA values for each of TAS, tumour parenchyma, and invasive front compartments of all patients.

Stromal average vasculature percentage (TAS-REA) was found at 0.91% when taking into consideration all patients. In tumour area the average value was 1.95% (T-REA) and in the invasive front was 4.2% of endothelial area (IF-REA). We noticed an increase of 2.15 times of relative vasculature area in the invasive front when compared to the tumour center. This observed trend confirmed our supposition that domain specific measurement may reveal more localized information about tumour angiogenesis development than whole slide parameters (see Figure 4, Table 1).

**Figure 5:**
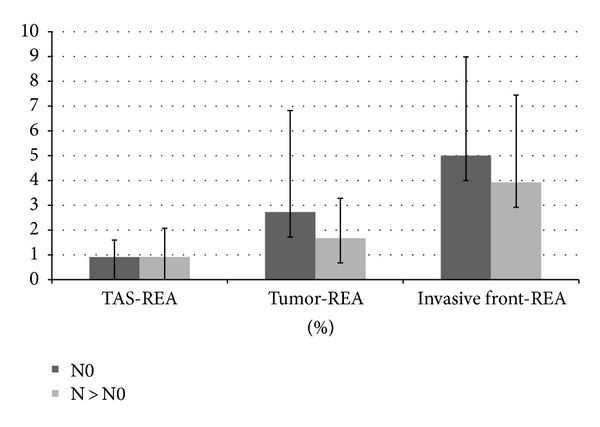
TAS-REA, T-REA, and IF-REA for both N0 and N > N0 group of patients. N0 group developed slightly more vasculature in tumour and invasive front compartments when comparing with N > N0 group.

Furthermore, similar statistical analysis was performed for each subgroup of lymph node negative (N0) patients as well as lymph node positive patients (N > N0). Same trends were observed for the three compartments in both groups: 0.91 in TAS, 2.72 in T, and 4.99 in IF for the N0 group and 0.92, 1.67, and 3.92 for the N > N0 group, respectively. This shows an increase of vasculature in the invasive front when compared to tumour or stroma values. However, we noticed that this trend is more pronounced in the N0 group of patients than in N > N0 group (4.99 versus 3.92, resp.) (see Figure 5).

### 3.2. Statistical Analysis

Statistical comparison of tumour compartments endothelial areas (TAS-REA, T-REA, and IF-REA) determined the following Pearson correlations and statistical differences. We found a positive weak statistical correlation but significant between TAS-REA and T-REA (*r* = 0.418, *P* = 0.003) and between TAS-REA and IF-REA (*r* = 0.432, *P* = 0.02) (see Table 2).

Two tailed *t*-test correlation analysis between tumour REA (T-REA) and the invasive front REA (IF-REA) showed a Pearson *r*-value of 0.655 and a *P*-value smaller than 0.001 (see Table 2). This highly significant moderate positive correlation shows that IF-REA has most likely the same trend as the T-REA: higher scores in tumour area are correlated with higher scores of invasive front REA.

When performing *t*-test correlation analysis for N0 and N > N0 groups, we noticed *r* = 0.851 (*P* < 0.001) and *r* = 0.565 (*P* < 0.001), respectively. Both groups showed a highly significant correlation between the two compartments (tumour and invasive front), with a higher correlation in the N0 group than in the N > N0 group.

Two-tailed *t*-test correlation analysis grouping by molecular subtypes revealed moderate to highly significant positive correlations only between some tumour compartments (see italic lines in Table 3), while for the rest no relevant correlations were found. The low number of patients within some molecular subtypes indicates the need of a larger study focused on such specific categories.

## 4. Discussions

Tumour angiogenesis became a point of interest for medical researchers since 1970 when Judah Folkman formulated the axiom that no tumour (new) tissue can grow more than 1-2 mm without development of new vasculature [1–5]. The process of angiogenesis is involved not only in tumour growth but also in metastasis development thus making its quantification a necessity [39–41]. In 1991, Weidner et al. was the first study to introduce the idea of microvessel density for assessment of tumour angiogenesis [7, 8]. This technique quantified the most vascularized tumour areas, the so called “hot spots” of the whole tumour. Later on, Chalkley graticule was introduced for easier counting, a technique which reduced interobserver variability and provided more reliable information about angiogenesis. Even so, the results of blood vessels counting were exposed to human error [6]. 

### 4.1. Methodological Comparison with Previous Work

As MVD and Chalkley techniques were extensively used for angiogenesis assessment soon after their introduction, newer studies improved the observer independence by enhancing the methods with various computer-aided image analysis systems (CIAS). The methods build further on the two phases of the analysis: selection of hot spots and assessment of vasculature. Thus, various methods of hotspot selection used lower magnification objectives (i.e., 4x or 5x) and an image processing step which detected locations with higher densities of the endothelial marker. However, they were placed regardless of the tumour domain (tumour associated stroma, tumour center, or its invasive front). Some [42] even went for a random selection of the locations. As vasculature network heterogeneity is extremely high in tumours, the location of such hot spots could have been in any of the three mentioned domains, albeit it is known that higher densities are to be expected in the invasive front. This step of hot-spot selection plays a crucial role in the method as also emphasized in [20], generating a high interstudy variability of results. Our work aimed at restricting the focus of the analysis based on these specific tumour compartments, which are known to exhibit distinct molecular, functional, structural, maybe even genetic heterogeneity. Other studies [25, 29, 31–33, 42–45] improved the vasculature assessment by measuring the total endothelial area (EA) and/or other morphological parameters (i.e., perimeter of found vessels, blood vessel area including lumen, and so forth). Regarding this aspect, we followed the trend going for EA measurement. To our best knowledge, no study performed endothelial area comparisons between tumour domains. Due to intended differences between current work and existing approaches, a direct result comparison does not seem to be feasible. Therefore, a methodological comparison was summarized in Table 4 showing only some relevant studies.

### 4.2. Digital Imaging Outlook

As digital image processing algorithms will evolve, our approach could be further improved. The first phase of domain specific region delineation could be performed automatically by dedicated algorithms, thus improving the observer independence. Regarding the measurement phase, further more advanced morphological parameters could be investigated, that is, using fractal and syntactic structure analysis [46]. Using other multiple staining protocols (i.e., using immunofluorescence techniques), the image information provided could be enhanced, thus providing more input to advanced image processing techniques.

### 4.3. Endothelial Marker Selection

A major factor in angiogenesis assessment is represented by the selection of the vessel marker [47, 48]. At the beginning Weidner used the antibody against von Willebrand factor, although this antibody was found to be specific for lymph vessels as well [6]. Other studies count blood vessels identified with antibody against CD31 or CD34. Reports [6, 49, 50] showed that CD31 may react with fibroblasts or confirm staining failures. Some studies [6, 49, 50] comparing staining of all three markers conclude CD34 as being the most reliable marker for evaluation of angiogenesis in immunohistochemistry. Several studies [51–54] propose CD105 or endoglin (receptor for TGF-*β*), a new marker which seems to be more specific to new tumour vessel. FSHR was reported to stain vessels located only at tumour border [55]. In our study, we used CD34 as recommended in Uzzan meta-analysis [6]. 

### 4.4. Domain Specific Assessment

Since tumour angiogenesis is enhanced by chemical stimulations, microenvironment may play a critical role in development of vasculature. However, the microenvironment in various morphopathological domains (i.e., tumour associated stroma, tumour area, and invasive front) is known to have high variations of protein density patterns [34–36]. Therefore, our study implemented domain-specific analysis, in contrast to whole-slide quantification which cannot reveal variations within various sites of the same patient [30–32]. Neither hot-spot selection nor grids with random fields expose the variations based on morphological context. They may show higher local vessel densities, but these sites are not related to tumour site patterns and morphology. Our approach separates morphologically different sites and performs local measurements specifically for these sites, revealing statistically relevant differences between them. In our study, hot-spot circles of the same area were set by a pathologist taking into consideration specific morphological properties of each area of interest. Splitting the tumour section in particular zones of interest brings not only higher measurement specificity but may help in developing stratification risk criteria as well, as it provides more detailed information than whole-section average measurements. 

### 4.5. Scanning Standardization

Digitization standardization was performed to achieve consistent image quality over the entire study. Virtual slides were realized with whole tissue sections so that any domain selection process is done having the entire imagistic morphological context available. Scanning only several sites selected directly on the microscope by one expert would have deprived the additional human experts from valuable unretrievable image data and would have dramatically restricted the secondary opinion analysis. In our case, the compartment selection phase was fully traceable and changeable during the reviewing process using virtual slide digital annotations.

### 4.6. Quantification Procedure

Our measurements aimed at cell-related parameters instead of targeting vasculature morphological entities typical to MVD count approaches. Therefore, we chose to measure REA of endothelial tissue in various morphopathological sites. This REA successfully characterizes total vasculature as well as provides a reliable base for endothelial proliferation index assessment considering that the area is statistically proportional with cell count. A comprehensive user validation phase was performed by looking at contours of found objects overlayed on the original images. Other image-type results (masks, gray level representations, and so forth) available in the software were also used. This approval phase took into consideration the expected morphology of the vasculature structures and allowed manual removal of falsely found areas which did not resemble blood vessels (i.e., high background, folded tissue, and so forth). Worth to mention that CD34 is known to stain also fibroblasts, which were also manually removed from analysis when found (*n* = 2). Out of 50 samples, 4 needed manual intervention.

### 4.7. Result Interpretation

After processing the data from quantification of endothelial area of interest, we noticed a significant difference of CD34 in tumour (T-REA = 1.95%) versus stroma (S-REA = 0.91%). The tumour has a vascularisation index 2.14 times higher than the tumour associated stroma index (*t*-test significance *P* < 0.0027). The invasive front REA was found at 4.2%, which is 2.15 times higher than the tumour REA index (*P* < 0.001) and 4.61 times higher than S-REA index (*P* < 0.002). These confirmed the expectations that the invasive front is more vascularized than the tumour parenchyma, which in turn has more vessels than the TAS. It is likely that an abundant vascularization at the periphery of the tumour (IF) may contribute to an increasing of the invasiveness of the tumour in the adjacent nontumoural tissue. However, this trend is slightly different in lymph node-negative and node-positive subgroups. Thus, in the node-negative group, the IF has a vascularization index 1.83 times higher than the corresponding tumour areas. In lymph node positive group, the same ratio was found to be 2.34, despite having both T and IF values lower than in the N0 subgroup. 

Various hot spots based measurements typically assessed with MVD may reveal local densities that are much higher than normal tissue, thus illustrating its malformed organization and highly heterogeneous network [56]. However, existing studies confirmed that the mean vascularization in tumours (lung, breast, kidney, and colon) is lower than in their normal counterparts [57], mainly due to proliferation being higher in tumour epithelial compartment than the endothelial one. Our results are also in trend with these findings, showing that aggravating stages tend to decrease overall average vasculature. Further studies focused on individual subgroups expressed in tumour molecular and genetic heterogeneity could reveal more insight on angiogenesis particularities.

When analysing the vascularisation according to molecular type of the tumours, we observed that in luminal A type carcinomas the statistical correlations for both pairs of compartments (T-IF and TAS-T) had moderate significance. This suggests that it might be a correlation between the better prognosis of the luminal A type breast carcinoma and the vascularization. On the other hand, for the rest of the molecular types (luminal B, basal-like, and HER2) we found highly significant positive correlations between some pairs of the three compartments. The correlations of different compartment pairs were not consistent between all molecular subtypes. This indicates different vasculature dynamism in each case since distinct variables and mechanisms may be involved. Correlations with other additional molecular signatures should be investigated in larger studies for a better understanding of the found differences. 

### 4.8. Clinical Relevance

The variations of REA index found between different tumour compartments, as well as between patient subgroups (lymph node negative versus node positive groups), show that domain specific REA measurements are capable of confirming and revealing additional important information about cancer development. Moreover, it may be an important criterion for further subgrouping and classification within already widely accepted histological scores. Thus, once new targets for cancer treatment are discovered, the proposed method can be used for assessment of the patient outcome. 

## 5. Conclusions

Relevant improvements in traceability and observer independence were realized by digitization of whole slide and virtual annotation of the domains of interest. The proposed measurement of relative endothelial area index for each of the tumour compartments (tumour associated stroma, tumour parenchyma, and invasive front) showed relevant differences in microvessel local density. It also showed differences between patients with or without lymph nodes metastases. The new digital scoring procedure can provide a precise measurement tool that promotes marker identification and correlation with a significant impact for patient management and eventually treatment individualization. By combining the experience of the pathologist in hot-spot selection with the precise measurement of the image processing approach, the proposed methodology brings new insights in clinical diagnostic, patient treatment, and follow-up evaluation.

## Figures and Tables

**Table 1:** REA values for each compartment (TAS, T, and IF) for all patients, lymph node negative subgroup (N0), and node positive subgroup (N > N0).

REA per compartment (relative endothelial area)	All patients, 50 cases	Node negative breast cancer patients (N0 group), 13 cases	Node positive breast cancer patients (N > N0 group), 37 cases
TAS%	0.91%	0.91%	0.92%
T%	1.95%	2.72%	1.67%
IF%	4.2%	4.99%	3.92%

**Table 2:** Paired samples correlations between tumour compartments in our study lot.

Pair of compartments	No. of cases	Pearson correlation		Statistic difference Sig (2-tailed) *P *
		*r *	Statistical significance 2-tailed *P *	
TAS-T	50	0.418	0.003 *P* < 0.01	0.002 *P* < 0.01
TAS-IF	50	0.432	0.002 *P* < 0.01	0.000 *P* < 0.001
T-IF	50	0.655	0.000 *P* < 0.001	0.000 *P* < 0.001

**Table 3:** Statistical correlations in different tumour compartments (TAS, T, and IF) within breast cancer molecular subtypes.

Molecular subtype of breast cancer	No. of cases	Pair of compartments	Correlation (*r*)	Statistical significance 2-tailed *P *
Luminal A	25	*TAS-T *	*0.426 *	*P = 0.034 P < 0.05 *
		TAS-IF	0.284	*P* = 0.169 *P* > 0.05
		*T-IF *	*0.499 *	*P = 0.011 P < 0.05 *

Luminal B	9	TAS-T	0.315	*P* = 0.410 *P* > 0.05
		TAS-IF	0.602	*P* = 0.086 *P* > 0.05
		*T-IF *	*0.862 *	*P = 0.003 P < 0.01 *

Basal-like	5	*TAS-T *	*0.966 *	*P = 0.007 P < 0.01 *
		TAS-IF	0.425	*P* = 0.475 *P* > 0.05
		T-IF	0.640	*P* = 0.244 *P* > 0.05

HER2	11	TAS-T	0.373	*P* = 0.258 *P* > 0.05
		TAS-IF	0.263	*P* = 0.434 *P* > 0.05
		*T-IF *	*0.912 *	*P = 0.000 P < 0.001 *

**Table 4:** Steps in evolution of vasculature assessment.

Study	Region measured	Parameter recommended	Measured by
Weidner et al. 1991 [7]	Hot spot at tumor border	MVD	human expert
Barbareschi et al. 1995 [25], Simpson et al. 1996 [43], Schoell et al. 1997 [44], Fridman et al. 2000 [45]	Hot spots identified on low magnification	EA, MVD	CIAS
Belien et al. 1999 [29]	Whole slide + hotspot	MVD	CIAS
Oh et al. 2001 [42]	Random spots	MVD	CIAS
Kim et al. 2003 [31]	Whole slide + hotspot	MVD + EA	CIAS
Chantrain et al. 2003 [32]	Entire sample	EA	CIAS
Mikalsen et al. 2011 [33]	Hot spots identified on low magnification	MVD	CIAS
Our method	Whole slide + domain specific large hot spots	EA	CIAS + human expert
